# The interplay between mesenchymal stem cells and the immune microenvironment in rotator cuff tendon-to-bone healing: current progress and future directions

**DOI:** 10.3389/fimmu.2025.1661340

**Published:** 2025-09-05

**Authors:** Dong Li, Yujia Zou, Yuanting Zhao

**Affiliations:** Day Surgery Center, Honghui Hospital Affiliated to Xi'an Jiaotong University, Xi’an, China

**Keywords:** rotator cuff tear, tendon-to-bone healing, mesenchymal stem cells, immune microenvironment, immunomodulation, tissue regeneration

## Abstract

Rotator cuff injuries frequently result in poor tendon-to-bone healing due to the failure to regenerate the native fibrocartilaginous enthesis and the persistence of a dysregulated immune microenvironment. Mesenchymal stem cells (MSCs) have emerged as promising therapeutic agents, not only for their multilineage differentiation potential but also for their potent immunomodulatory functions. Emerging evidence highlights that MSCs engage in bidirectional crosstalk with immune cells such as macrophages, T cells, and NK cells through both paracrine factors and direct cell–cell contact, critically shaping the reparative versus fibrotic outcome of tendon-to-bone healing. This review summarizes the biological mechanisms underlying MSC-mediated tendon-to-bone healing, with a focus on immune modulation. We discuss recent advances in cell-free approaches, biomaterial-assisted delivery systems, and strategies to enhance the local immune milieu. Current challenges—including MSC heterogeneity, variable patient immune responses, and translational barriers—are also addressed. Finally, we highlight future directions such as personalized immunomodulatory therapies, 3D humanized testing models, and AI-based prediction tools aimed at improving clinical outcomes. Specifically, AI algorithms that integrate patient-specific immune profiles—such as single-cell transcriptomics and cytokine signatures—may enable responder stratification and guide individualized MSC-based interventions. Understanding and leveraging the MSC–immune interaction is key to unlocking the full potential of regenerative therapies in rotator cuff repair.

## 1 Introduction

Rotator cuff tears are one of the most common causes of shoulder dysfunction and chronic pain, affecting millions of people worldwide. In the United States alone, over 460,000 rotator cuff repair surgeries are performed annually, underscoring the significant clinical and socioeconomic burden of this condition (1). Despite advances in surgical techniques and materials, postoperative tendon-to-bone healing remains suboptimal, with reported retear rates ranging from 29.5% to as high as 94% in some populations (2, 3). Such high failure rates significantly compromise patient outcomes and long-term quality of life.

A key biological obstacle lies in the inability to regenerate the native fibrocartilaginous enthesis, a highly specialized transition zone between tendon and bone. Instead, surgical repair commonly results in fibrous scar tissue formation at the tendon-bone junction, which lacks the zonal organization and mechanical strength of the native interface, making the repair site susceptible to mechanical failure (4, 5).

Moreover, emerging evidence suggests that dysregulation of the local immune microenvironment and persistent inflammation also play a critical role in impairing the quality of tendon-to-bone healing (6). Following injury and surgical repair, the recruitment and activation of immune cells such as macrophages, T cells, and neutrophils initiate an inflammatory cascade that may either support or hinder regeneration, depending on the balance between pro-inflammatory and pro-regenerative signals (7).

In recent years, cell-based therapies—particularly those involving mesenchymal stem cells (MSCs)—have gained attention for their multifaceted role in musculoskeletal regeneration. MSCs are multipotent stromal cells capable of differentiating into osteogenic, chondrogenic, and tenogenic lineages. Beyond differentiation, MSCs exert profound immunomodulatory and paracrine effects by secreting anti-inflammatory cytokines and regenerative growth factors such as TGF-β, VEGF, and IL-10 (8, 9). These properties enable MSCs to reshape the immune microenvironment, reduce excessive inflammation, and facilitate the formation of a more functional enthesis-like structure.

Furthermore, MSC-derived extracellular vesicles (EVs), conditioned media, and scaffold-based delivery systems have been explored as cell-free strategies to harness the regenerative potential of MSCs while minimizing the risks associated with direct cell transplantation (10, 11). In parallel, immunomodulatory strategies—such as promoting M2 macrophage polarization or inhibiting excessive M1 activation—have shown promise in improving tendon-to-bone healing outcomes (12).

This review aims to provide a comprehensive overview of the current understanding of MSCs and their interaction with the immune microenvironment in the context of rotator cuff tendon-to-bone healing. We focus on the biological mechanisms underlying their crosstalk, highlight recent advances in preclinical and translational research, and discuss future directions for biomaterial-assisted, immune-responsive, stem cell-based therapies.

## 2 Biological basis of tendon-to-bone healing in rotator cuff repair

### 2.1 Native enthesis structure and healing response

#### 2.1.1 Four-zone interface: tendon, fibrocartilage, mineralized fibrocartilage, and bone

**Figure 1:**
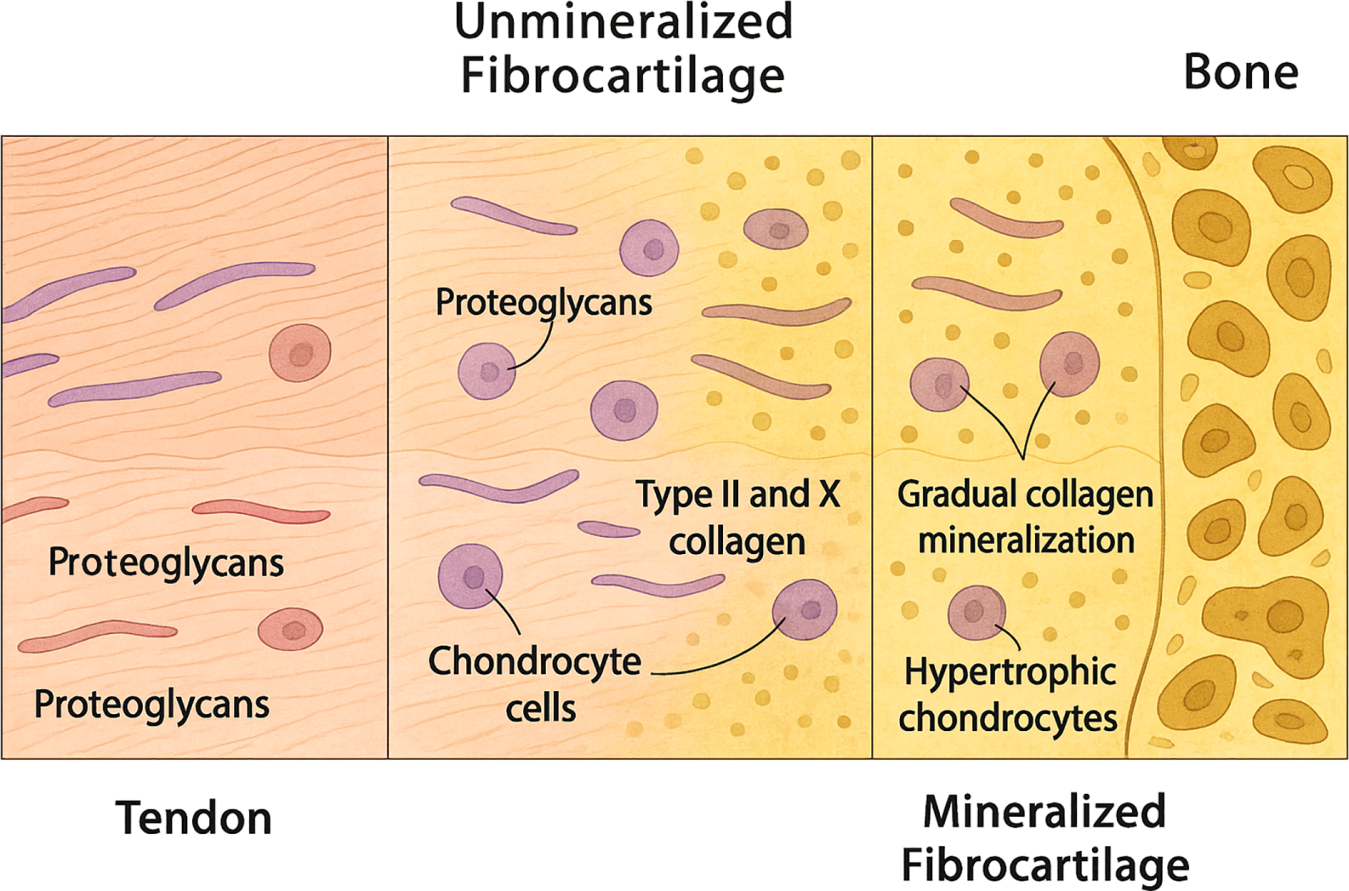
A schematic representation of the tendon-to-bone interface, depicting the histological characteristics of its four transitional zones.

The tendon-to-bone interface of the rotator cuff, or enthesis, is a specialized structure that facilitates load transfer between tendon and bone. As **Figure 1** depicts, it comprises four distinct zones: (1) tendon, (2) unmineralized fibrocartilage, (3) mineralized fibrocartilage, and (4) bone (13–15).

Each zone features unique cells, matrix components, and mechanical roles:

Zone 1 – Tendon: Rich in aligned type I collagen and tenocytes, this region offers strong tensile resistance (13).
Zone 2 – Unmineralized Fibrocartilage: Contains type II collagen, proteoglycans, and chondrocyte-like cells, helping to absorb and spread mechanical stress (13, 16).
Zone 3 – Mineralized Fibrocartilage: Shows gradual collagen mineralization with type II and X collagen and hypertrophic chondrocytes, forming a transitional zone for stress transfer (14, 15).
Zone 4 – Bone: Composed of mineralized matrix and osteocytes, providing structural anchoring (14).

This four-zone architecture develops after birth, with collagen and cell phenotypes gradually establishing a spatial gradient along the interface (14, 15). This organization ensures efficient load transmission and mechanical durability.

Following injury and repair, this native structure is rarely restored. The interface is often replaced by disorganized fibrous scar tissue lacking zonal features, with disrupted collagen alignment, reduced chondrocyte presence, and impaired mechanical strength (13, 17, 18). Animal studies show that the regenerated interface typically resembles a single fibrous layer with limited function (15, 17, 18).

Recent tissue engineering strategies have attempted to replicate the zonal structure using growth factors, scaffolds, and stem cells. Some animal models have achieved partial regeneration of multilayered tissue with improved outcomes, but clinical success remains limited (14, 16, 19).

The absence of native enthesis architecture remains a major barrier to durable repair.

#### 2.1.2 Inadequate reformation after injury leading to biomechanical weakness

Following rotator cuff injury and surgical repair, the restoration of the native tendon-to-bone interface (enthesis) is rarely achieved. Instead, healing is typically characterized by the formation of disorganized fibrovascular scar tissue, which lacks the highly specialized zonal architecture and mineralized fibrocartilage of the native enthesis (20, 21). This aberrant repair tissue exhibits inferior mechanical properties, including reduced load-to-failure and decreased stiffness, predisposing the repair site to retear under physiological loading (1, 15).

Histological and ultrastructural analyses reveal that the repaired interface fails to recapitulate the gradation of collagen types, mineral content, and cellular morphology seen in the normal enthesis (16, 17). The absence of these gradients results in a biomechanical mismatch between tendon and bone, further compromising the integrity of the repair (17, 21). Animal studies consistently demonstrate that the newly formed interface is mechanically weaker than the native insertion, with lower ultimate tensile strength and increased susceptibility to failure (22, 23).

Current repair techniques, including suture and anchor-based methods, do not adequately address the biological requirements for enthesis regeneration, often resulting in persistent biomechanical deficits (1, 22). While scaffold augmentation and biologic strategies such as growth factor delivery and stem cell incorporation have shown promise in preclinical models for enhancing fibrocartilage formation and improving mechanical strength, translation to consistent clinical benefit remains limited (3, 24). Thus, inadequate reformation of the enthesis after injury remains a principal cause of biomechanical weakness and high retear rates following rotator cuff repair (15, 17).

### 2.2 Phases of tendon-to-bone healing

**Figure 2:**
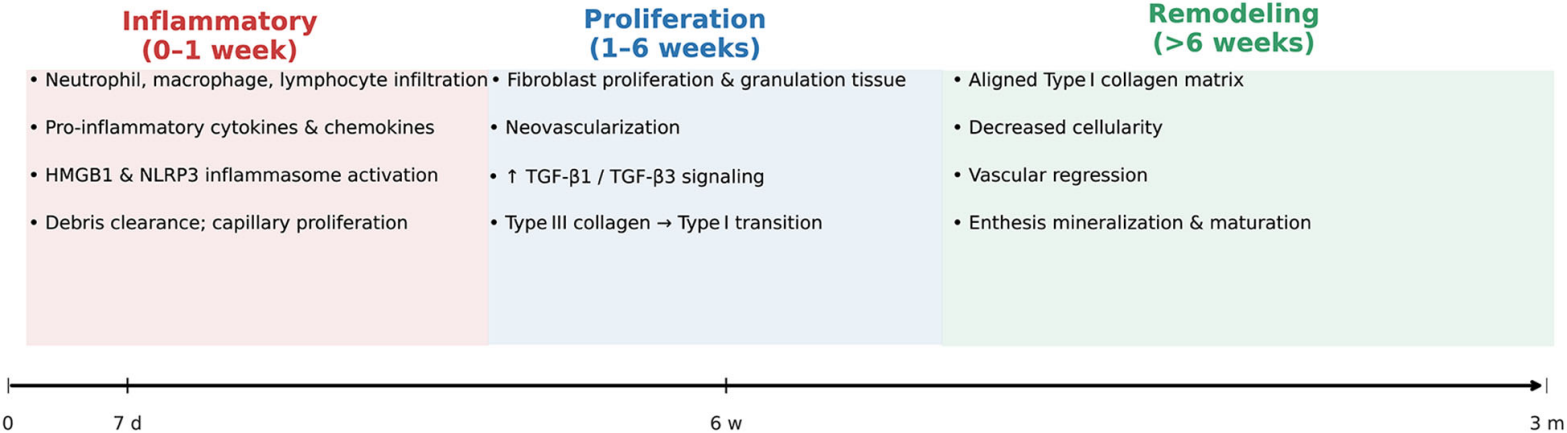
Temporal Phases of Tendon-to-Bone Healing After Rotator Cuff Repair. This schematic timeline illustrates the sequential inflammatory (0–1 week), proliferative (1–6 weeks), and remodeling (>6 weeks) phases of rotator cuff tendon-to-bone healing. Key cellular activities (e.g., immune-cell infiltration, fibroblast proliferation, collagen remodeling) and principal molecular signals (HMGB1/NLRP3, TGF-β1/β3) are annotated within each phase, highlighting the transition from an early inflammatory milieu to organized, mineralizing enthesis tissue.

Tendon-to-bone healing after rotator cuff repair is classically divided into three overlapping phases (**Figure 2**): the inflammatory phase, the proliferation phase, and the remodeling phase (20). Each phase is characterized by distinct cellular and molecular events that ultimately determine the quality of the repair tissue and its biomechanical properties (25, 26).

#### 2.2.1 Inflammatory phase

Immediately after tendon reattachment, the healing site undergoes an acute inflammatory response. Neutrophils, macrophages, and lymphocytes infiltrate the repair interface, releasing pro-inflammatory cytokines and chemokines that initiate debris clearance and recruit reparative cells (27). Upregulation of HMGB1 and activation of the NLRP3 inflammasome have been implicated in amplifying inflammation and extracellular matrix (ECM) disorganization during this period. Capillary proliferation and increased cellularity are observed within the first week, peaking around 7–10 days post-repair. This early inflammatory milieu is essential for subsequent healing, but excessive or prolonged inflammation can impair matrix organization and enthesis regeneration (28, 29).

#### 2.2.2 Proliferation phase

The proliferative phase is marked by robust fibroblast proliferation, neovascularization, and synthesis of new ECM, predominantly type III collagen. Growth factors such as TGF-β1 and TGF-β3 are upregulated, promoting cell proliferation and matrix deposition. This phase typically spans from the end of the first week to several weeks post-injury (27, 30). The repair tissue remains highly cellular and disorganized, with ongoing angiogenesis and granulation tissue formation. The transition from type III to type I collagen synthesis begins during this phase, setting the stage for subsequent tissue maturation (27, 31).

#### 2.2.3 Remodeling phase

During the remodeling phase, which can extend from several weeks to months, the initially disorganized collagen matrix is gradually replaced by more organized, aligned type I collagen fibers. Cellular density decreases, and the vascular network regresses (20, 27). The enthesis undergoes slow maturation, with partial restoration of zonal architecture and mineralization, although the regenerated interface rarely achieves the structural and biomechanical properties of the native enthesis. The ratio of type I to type III collagen increases, and the tissue becomes more resistant to mechanical loading, but persistent differences in organization and strength compared to uninjured tendon-bone insertions remain (30, 31).

Overall, the incomplete recapitulation of the native enthesis structure and the persistence of scar-mediated healing contribute to the biomechanical weakness and high retear rates observed after rotator cuff repair (27, 31).

### 2.3 Role of immune cells in tendon-to-bone healing

**Figure 3:**
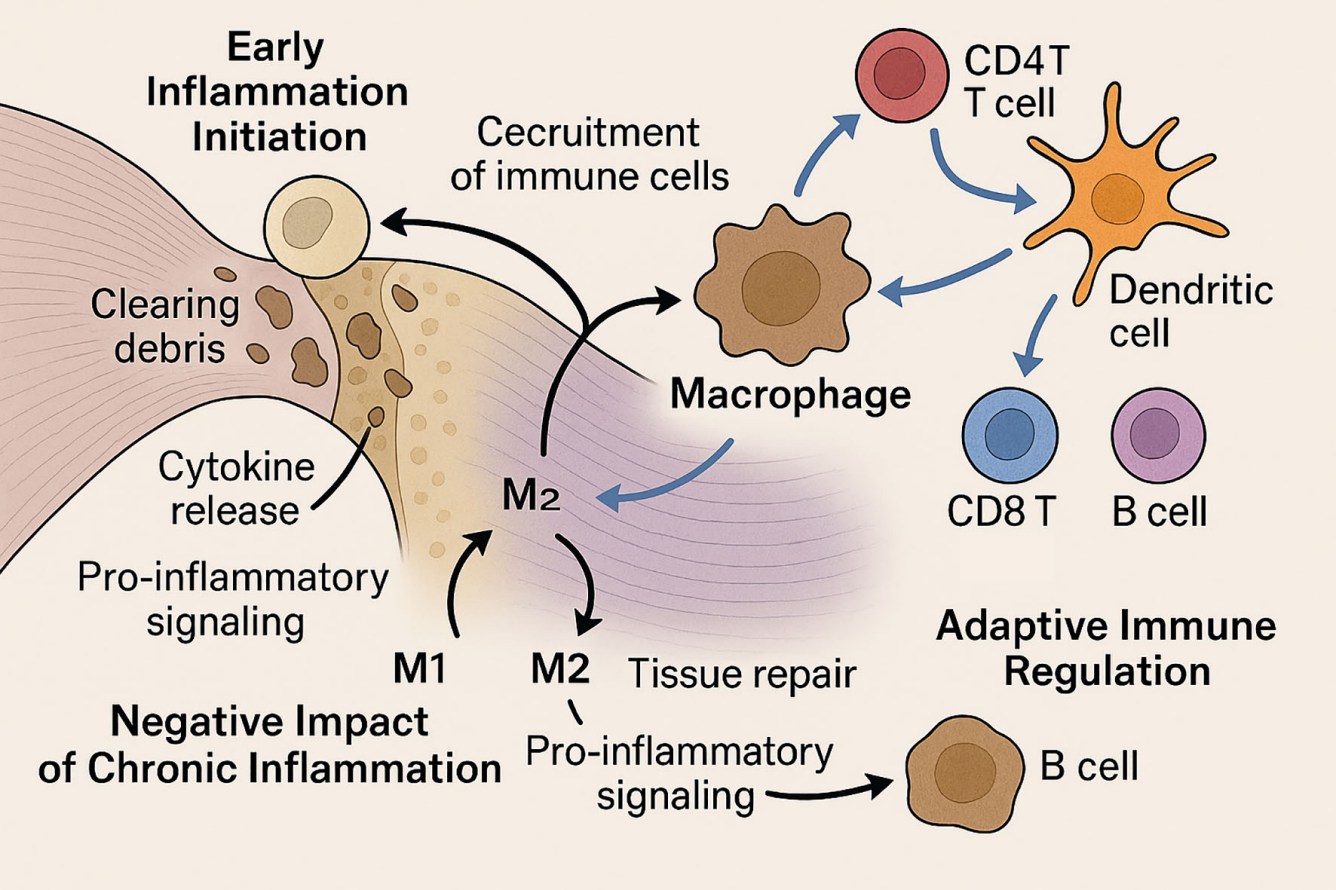
Immune Cell Dynamics in Rotator Cuff Tendon-to-Bone Healing. This schematic illustration depicts the roles of immune cells in rotator cuff tendon-to-bone healing across different stages. In the early phase, neutrophils are the first responders, clearing debris and releasing cytokines to recruit additional immune cells. Macrophages follow, mediating phagocytosis and orchestrating the inflammatory response. Chronic inflammation, driven by sustained M1 macrophage activity, leads to excessive scarring and impaired matrix organization. In contrast, M2 macrophage polarization promotes tissue repair. During later phases, adaptive immune cells such as CD4^+^ and CD8^+^ T cells, B cells, and dendritic cells accumulate at the healing site and coordinate with macrophages to modulate inflammation and facilitate tissue remodeling. This coordinated immune regulation is critical for optimal tendon-to-bone integration.

Below, we provide a detailed overview of how both innate (neutrophils, macrophages) and adaptive (T and B lymphocytes, dendritic cells) immune cells coordinate the inflammatory, proliferative, and remodeling phases of tendon-to-bone healing; their dynamic interplay is schematically summarized in **Figure 3**.

#### 2.3.1 Neutrophils and macrophages initiate inflammation

Following rotator cuff injury and repair, neutrophils are among the first immune cells to infiltrate the tendon-to-bone interface, rapidly clearing debris and releasing cytokines that recruit additional immune cells (32). Macrophages follow, performing phagocytosis, secreting growth factors, and orchestrating the early inflammatory response (33). Both tendon-resident and circulating macrophages are involved, with chemokine receptor CCR2 playing a critical role in their recruitment and function (34). These early innate immune responses are essential for debris clearance and the initiation of tissue repair, but their magnitude and duration must be tightly regulated (35).

#### 2.3.2 Chronic inflammation impairs interface regeneration

While acute inflammation is necessary for healing, persistent or dysregulated inflammation impairs tendon-to-bone interface regeneration (36, 37). Prolonged macrophage activation and sustained pro-inflammatory signaling can lead to excessive scar formation, extracellular matrix disorganization, and biomechanical weakness at the repair site (38). The balance between pro-inflammatory (M1) and anti-inflammatory (M2) macrophage phenotypes is particularly important; a shift toward M2 polarization is associated with improved healing outcomes, while persistent M1 activity is linked to fibrosis and poor integration (39). Biologic adjuvants and stem cell-derived exosomes have been shown to promote M2 polarization and attenuate chronic inflammation, thereby enhancing tendon-to-bone healing (40).

#### 2.3.3 T cells and dendritic cells in adaptive immune modulation

Adaptive immune cells, including T cells and dendritic cells, accumulate at the repair site and in draining lymph nodes during the subacute and remodeling phases (33, 34). CD4+ T cells and dendritic cells coordinate the immune response, influence macrophage polarization, and regulate the transition from inflammation to tissue remodeling (36, 37). B cells and CD8+ T cells also increase over time, suggesting a sustained adaptive response that may impact long-term healing quality. The coordinated actions of these immune cell populations are essential for optimal tendon-to-bone healing, and dysregulation at any stage can compromise repair integrity (40).

## 3 Mesenchymal stem cells in tendon-to-bone healing

### 3.1 Sources and properties of MSCs

The common types of MSCs, including those derived from bone marrow, adipose tissue, synovium, and tendon, along with key features are summarized in **Table 1**.

**Table 1:** Comparison of MSC sources and their properties.

MSC source	Multipotency	Proliferative capacity	Immunomodulatory function	Advantages/notes
Bone marrow-derived MSCs (BM-MSCs)	Osteogenic, chondrogenic, tenogenic	Moderate	Strong: regulates macrophage polarization, suppresses inflammation (41)	Extensively studied; secrete trophic factors promoting repair (42, 43)
Adipose-derived MSCs (AD-MSCs)	Comparable to BM-MSCs	High	Strong: secretes anti-inflammatory cytokines (2, 44)	Easily accessible, large yield, minimally invasive (45)
Synovial-derived MSCs	Chondrogenic potential	High	Modulates macrophage polarization (46)	Tissue-specific; promising for cartilage-related repair (46)
Tendon-derived MSCs	Tenogenic lineage	Moderate	Suppresses pro-inflammatory signaling (46)	Enhances tendon matrix integration; tissue-specific (47)

#### 3.1.1 Bone marrow-derived MSCs

BM−MSCs are most used; they show tri−lineage differentiation and trophic, proangiogenic, and immunoregulatory (anti−inflammatory, macrophage−polarizing) effects that support tendon–bone healing (42, 43, 48).

#### 3.1.2 Adipose-derived MSCs

AD−MSCs are high−yield and minimally invasive (43), retain BM−MSC−like multipotency but greater proliferation and anti−inflammatory paracrine activity (2), aiding inflammation resolution, matrix remodeling, and interface regeneration (44).

#### 3.1.3 Synovial and tendon-derived MSCs

Synovial MSCs are highly proliferative/chondrogenic, and tendon−derived MSCs are tenogenic; both modulate macrophage polarization and curb pro−inflammatory signaling, favoring targeted tendon/enthesis repair (46).

#### 3.1.4 Characteristics: multipotency, trophic effects, immune regulation

Across sources, MSCs are multipotent, yielding tendon− and bone−relevant lineages. Through trophic factor/EV secretion they recruit cells, promote angiogenesis and ECM remodeling, and via immunomodulation (↓ pro−inflammatory, ↑ anti−inflammatory, immune−phenotype control) they establish a regenerative milieu that improves tendon–bone healing (44).

MSCs show source−dependent phenotypes and functions that shape regeneration. AD−MSCs are strongly immunomodulatory and tolerate inflammatory niches (49–51), whereas BM-MSCs have superior osteogenic capacity, supporting bone and enthesis repair (52, 53). Synovial MSCs are chondrogenic, while tendon−derived MSCs are tenogenic, aligning with cartilage and tendon repair, respectively (54–57). These traits likely drive outcome differences in preclinical models and support source–tissue–context matching.

### 3.2 MSC mechanisms of action

Mesenchymal stem cells (MSCs) have emerged as a promising therapeutic modality for enhancing tendon-to-bone healing, a process that remains clinically challenging due to the complex structure and limited regenerative capacity of the enthesis. The mechanisms by which MSCs facilitate this repair are multifactorial and involve both direct and indirect actions (44, 47, 48, 58).

#### 3.2.1 Differentiation into tenocytes, chondrocytes, osteoblasts

MSCs possess multilineage differentiation potential, enabling them to give rise to tenocytes, chondrocytes, and osteoblasts under appropriate microenvironmental cues (45, 58). This plasticity is critical for reconstructing the transitional zones of the tendon-bone interface, which require the regeneration of fibrocartilaginous and osseous tissues (59). *In vivo* and *in vitro* studies have demonstrated that MSCs can integrate into injured sites and differentiate along these lineages, contributing to the restoration of native tissue architecture and biomechanical properties (59, 60).

#### 3.2.2 Secretion of bioactive factors: TGF-β, VEGF, IGF-1, IL-6

Beyond differentiation, MSCs exert significant paracrine effects by secreting a repertoire of bioactive molecules, including transforming growth factor-beta (TGF-β), vascular endothelial growth factor (VEGF), insulin-like growth factor 1 (IGF-1), and interleukin-6 (IL-6) (58). These factors modulate the local microenvironment by promoting cell proliferation, suppressing inflammation, and enhancing extracellular matrix (ECM) synthesis. The MSC secretome, including exosomes, has been shown to influence tenocyte migration, angiogenesis, and matrix remodeling, further supporting tissue repair (8, 61).

#### 3.2.3 Promotion of angiogenesis and matrix remodeling

MSCs facilitate neovascularization at the repair site, primarily through the secretion of angiogenic factors such as VEGF. Enhanced vascularity is essential for nutrient delivery and waste removal, supporting the metabolic demands of regenerating tissues (46, 62). Additionally, MSCs promote ECM remodeling by upregulating the synthesis of type I collagen and other matrix proteins, and by modulating the balance between matrix metalloproteinases and their inhibitors, thereby improving the quality and organization of the repair tissue (8, 61).

#### 3.2.4 Recruitment and activation of endogenous progenitor cells

MSCs can recruit and activate endogenous progenitor cells through the release of chemotactic and immunomodulatory factors. This recruitment amplifies the regenerative response by increasing the pool of reparative cells at the injury site (46, 58). Furthermore, MSCs modulate the inflammatory milieu, creating a pro-regenerative environment that supports the survival and function of both transplanted and resident progenitor cells (59, 61).

Collectively, these mechanisms underscore the therapeutic potential of MSCs in tendon-to-bone healing. However, challenges remain regarding the optimization of cell source, delivery methods, and standardization of protocols to maximize clinical efficacy (47, 60, 63).

### 3.3 Preclinical and clinical evidence

#### 3.3.1 Overview of *in vivo* studies using MSCs in rotator cuff models

Recent preclinical studies have demonstrated that MSCs and their derivatives can significantly enhance tendon-to-bone healing in rotator cuff injury models. In rat and rabbit models, local delivery of MSCs—whether as cell suspensions, exosomes, or cell sheets—has been shown to improve fibrocartilage formation, collagen maturation, and biomechanical strength at the enthesis (64, 65). For example, exosomes derived from kartogenin-preconditioned MSCs or loaded with kartogenin have been shown to promote chondrogenesis, collagen organization, and superior biomechanical properties compared to controls (66). Menstrual blood-derived MSCs encapsulated in platelet-rich gel facilitated new bone and fibrocartilage formation, with improved mechanical properties in a rabbit chronic tear model (65). Similarly, umbilical cord MSC-derived extracellular vesicles and cryopreserved adipose-derived stem cell sheets have demonstrated efficacy in large animal and rabbit models, respectively, supporting the translational potential of off-the-shelf or cell-free approaches (7, 67).

Genetic modification of MSCs, such as Msx1 overexpression, has also been explored to enhance proliferation and migration, resulting in improved tendon-bone integration and mechanical strength in rat models (11). The use of biomaterial scaffolds, such as 3D-printed polycaprolactone loaded with basic fibroblast growth factor and MSCs, further augments osteogenesis and immunomodulation, leading to improved enthesis healing (68). Conditioned medium from human bone marrow-derived MSCs has been shown to promote tendon-bone healing via immunomodulation, particularly through macrophage polarization (8). Collectively, these studies provide strong mechanistic and functional evidence for the role of MSCs in enhancing tendon-to-bone healing in preclinical models.

#### 3.3.2 Clinical trials and limitations

Clinical translation of MSC-based therapies for rotator cuff repair remains in early stages, with limited but growing evidence from human studies. Early-phase clinical trials and case series suggest that MSC augmentation may reduce retear rates and improve structural outcomes after rotator cuff repair, but results are heterogeneous and often limited by small sample sizes, lack of standardized protocols, and short-term follow-up (2). Safety profiles are generally favorable, with no major adverse events reported in the available literature (2).

Key limitations include variability in MSC source (bone marrow, adipose, umbilical cord, menstrual blood), cell processing methods, delivery vehicles, and dosing regimens. Clinical heterogeneity in MSC-based therapies arises from several factors beyond cell source and delivery. Variations in surgical technique—such as single- vs. double-row repair and open vs. arthroscopic approaches—can affect MSC retention and local biomechanics, influencing treatment outcomes (69, 70). Patient-related variables including age, diabetes, smoking, and baseline inflammation also impact MSC responsiveness, with evidence that aging and metabolic disorders reduce MSC efficacy (71, 72). Differences in processing—such as cryopreservation, passage number, and preconditioning—as well as culture conditions contribute to product variability and functional inconsistency (72–74). These factors are rarely standardized across studies, complicating data comparison and contributing to inconsistent results. Harmonizing protocols and improving reporting practices are essential for enhancing reproducibility in MSC research.

Furthermore, the optimal timing, route of administration, and patient selection criteria remain to be defined. The lack of large, multicenter randomized controlled trials with standardized outcome measures and long-term follow-up precludes definitive conclusions regarding efficacy (64). Regulatory, ethical, and logistical challenges also hinder widespread clinical adoption. Ongoing research is focused on optimizing cell-free approaches (e.g., exosomes, conditioned medium), biomaterial scaffolds, and strategies to recruit endogenous MSCs, which may address some of these barriers and facilitate broader clinical translation (7, 8).

In summary, robust preclinical evidence supports the potential of MSC-based therapies to enhance tendon-to-bone healing in rotator cuff repair, but clinical evidence remains preliminary. Further high-quality, standardized clinical trials are needed to establish efficacy, safety, and best practices for MSC application in this context.

## 4 The immune microenvironment and its impact on MSC function

**Figure 4:**
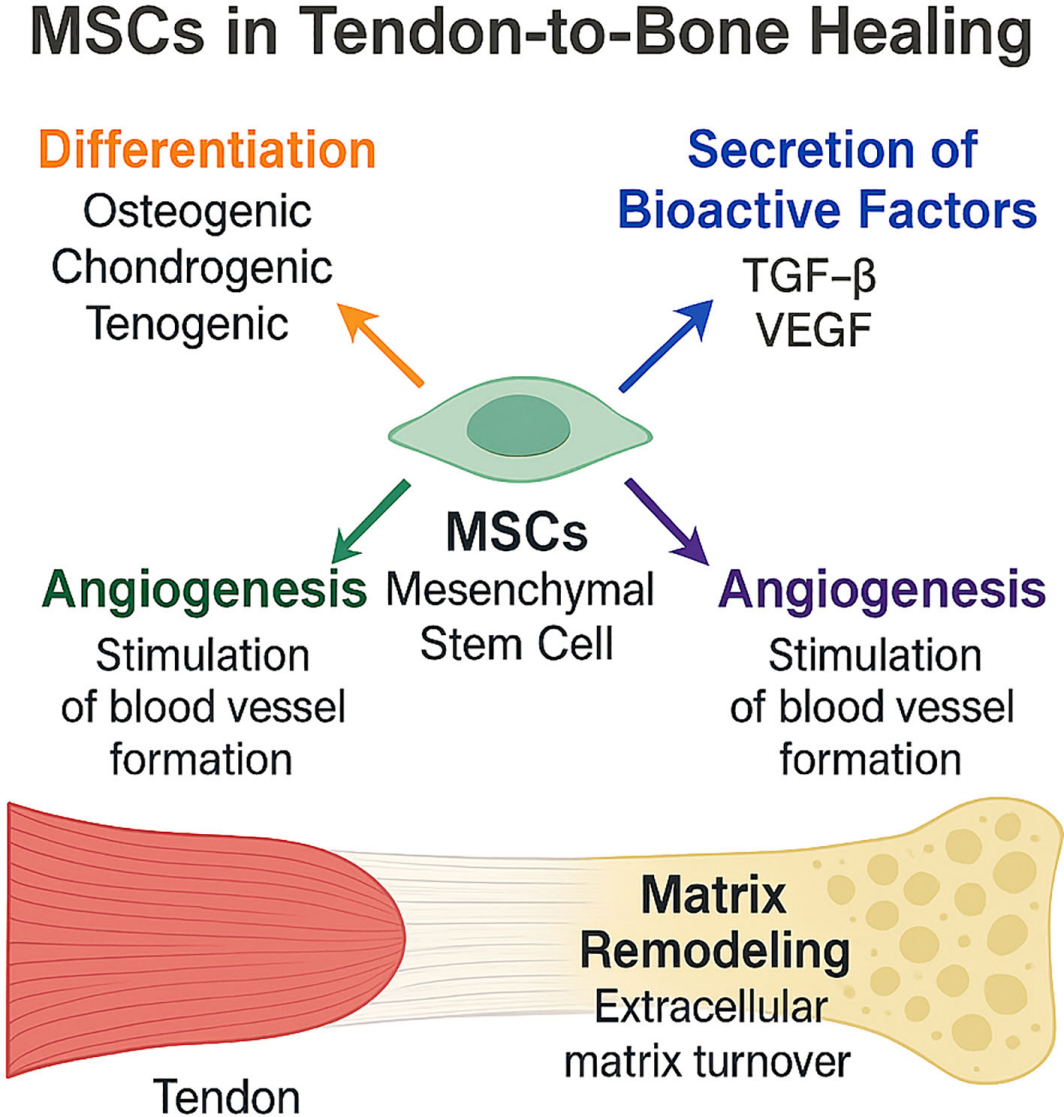
Mechanisms of Mesenchymal Stem Cell (MSC)-Mediated Tendon-to-Bone Healing. This schematic illustrates the multifactorial contributions of MSCs to rotator-cuff tendon-to-bone repair. MSCs differentiate toward osteogenic, chondrogenic, and tenogenic lineages; secrete bioactive factors such as TGF-β and VEGF; stimulate angiogenesis; and drive extracellular-matrix remodeling at the enthesis, collectively promoting robust tendon-bone integration.

The immune microenvironment at the tendon-to-bone interface following rotator cuff injury is a dynamic and complex milieu that critically influences the reparative capacity of mesenchymal stem cells (**Figure 4**). The interplay between immune cells, cytokines, and MSCs determines the balance between regeneration and fibrosis, ultimately impacting clinical outcomes after rotator cuff repair (2, 8, 38, 39).

### 4.1 The local immune landscape after rotator cuff injury

#### 4.1.1 Acute vs. chronic inflammation

Acute inflammation is characterized by a rapid influx of neutrophils and pro-inflammatory macrophages, which are essential for debris clearance and the initiation of tissue repair. However, a timely resolution of this response is necessary to prevent chronic inflammation, which is associated with persistent infiltration of inflammatory cells, elevated levels of pro-inflammatory cytokines, and impaired tendon-to-bone healing (37, 39). Chronic inflammation, often observed in aged or degenerative rotator cuff tears, leads to a fibrotic microenvironment that impairs MSC function and promotes scar tissue formation rather than regeneration (1, 38, 39).

#### 4.1.2 M1 vs. M2 macrophage polarization

Macrophages are central regulators of the immune microenvironment, exhibiting plasticity between the pro-inflammatory M1 phenotype and the anti-inflammatory, pro-regenerative M2 phenotype. M1 macrophages secrete high levels of tumor necrosis factor alpha (TNF-α) and interleukin-1 beta (IL-1β), promoting inflammation and tissue degradation. In contrast, M2 macrophages produce interleukin-10 (IL-10) and transforming growth factor beta (TGF-β), supporting resolution of inflammation, matrix remodeling, and tissue regeneration (8, 38, 42). The polarization state of macrophages at the tendon-bone interface is a key determinant of MSC fate and function. Recent studies demonstrate that MSC-derived secretomes and exosomes can shift macrophage polarization from M1 to M2, thereby creating a more favorable environment for tendon-to-bone healing (38, 39, 75).

#### 4.1.3 Pro-inflammatory (TNF-α, IL-1β) vs. anti-inflammatory (IL-10, TGF-β) cytokine profiles

The cytokine milieu at the injury site is a major modulator of both immune cell and MSC behavior. Elevated levels of TNF-α and IL-1β are hallmarks of the pro-inflammatory phase and are associated with impaired healing and increased fibrosis (38, 75). Conversely, anti-inflammatory cytokines such as IL-10 and TGF-β promote the resolution of inflammation, enhance MSC-mediated tissue repair, and support the transition to a regenerative microenvironment (8, 38, 39). The balance between these cytokine profiles is dynamic and can be therapeutically modulated. For example, administration of MSC-conditioned medium or exosomes has been shown to suppress pro-inflammatory cytokines and upregulate anti-inflammatory mediators, thereby improving tendon-to-bone healing in preclinical models (1, 8, 38, 75).

In summary, the immune microenvironment after rotator cuff injury is a critical determinant of MSC function and tendon-to-bone healing. Acute inflammation is necessary for initiating repair, but chronic inflammation impairs regeneration. The polarization of macrophages and the balance of cytokine profiles are central to this process, and therapeutic strategies that modulate these factors—such as MSC-derived secretomes—hold promise for improving clinical outcomes (1, 8, 38).

### 4.2 Crosstalk between MSCs and immune cells

**Figure 5:**
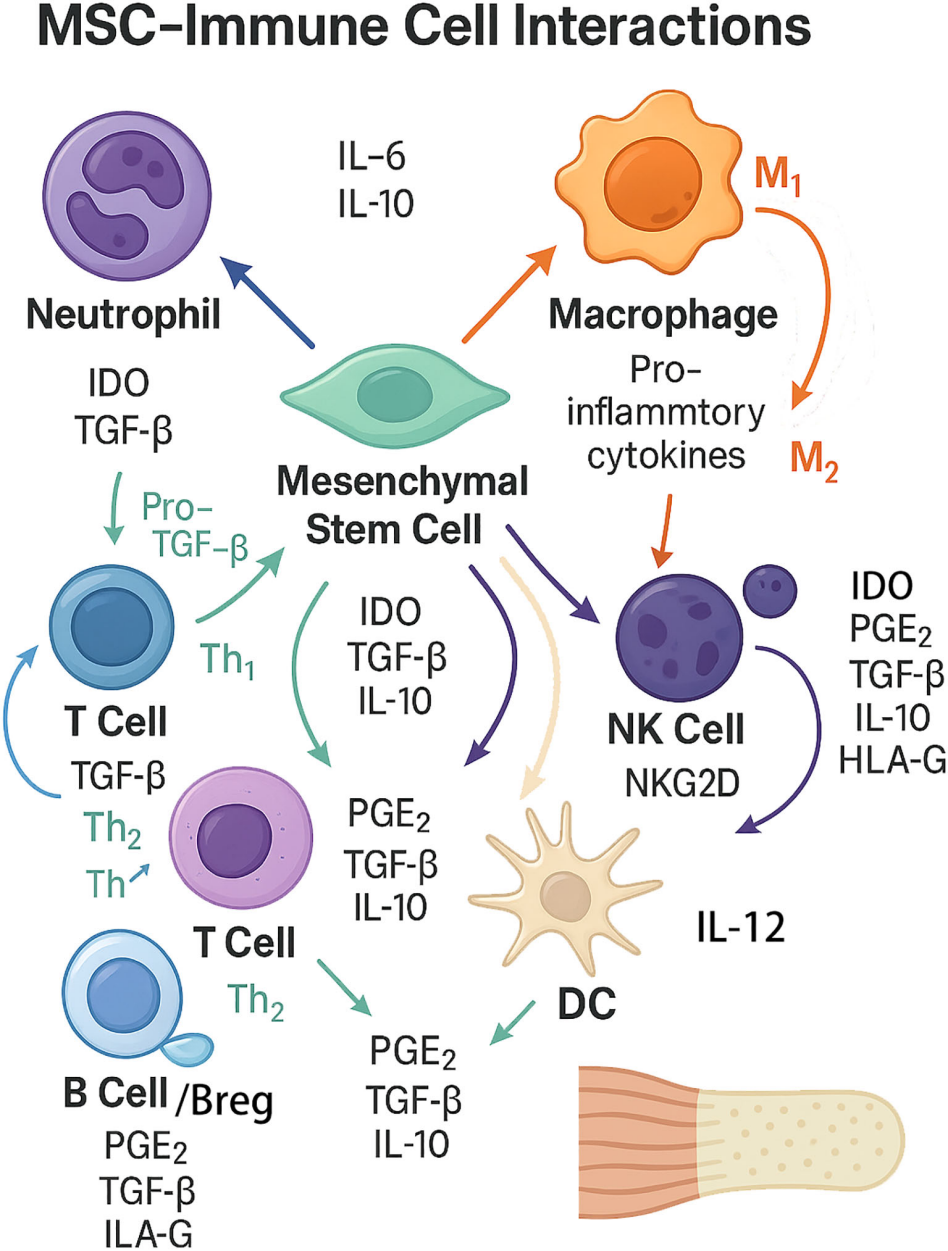
The complex bidirectional interactions between mesenchymal stem cells (MSCs) and key immune cell types involved in tendon-to-bone healing. The diagram summarizes bidirectional interactions between mesenchymal stem cells (MSCs) and innate/adaptive immune cells. MSCs suppress neutrophil−driven inflammation and modulate macrophage polarization toward an M2, pro−repair phenotype through PGE_2_, TGF−β, IL−10, and IDO, with auxiliary signals HLA−G and TSG−6; MSC extracellular vesicles (EVs) further reinforce anti−inflammatory signaling. MSCs inhibit effector T cells while favoring Treg/Th2 responses (IDO–kynurenine pathway, NO, TGF−β, PGE_2_, HLA−G, TSG−6), attenuate NK cell activation and cytotoxicity (PGE_2_, TGF−β, IL−10, IDO, HLA−G; NKG2D pathway), restrain dendritic cell maturation and IL−12/co−stimulation to promote tolerogenic DCs, and limit B−cell proliferation/differentiation while inducing IL−10^+^ Breg cells. Conversely, inflammatory cues from immune cells—especially TNF−α and IFN−γ from macrophages/T cells—can “license” MSCs or, when excessive, inhibit MSC survival and osteo/tenogenic differentiation. This coordinated immunoregulation mitigates excessive inflammation and supports matrix remodeling and enthesis integration.

The crosstalk between mesenchymal stem cells (MSCs) and immune cells is central to tendon-to-bone healing after rotator cuff injury (**Figure 5**) (8, 76). MSCs exert immunomodulatory effects through both direct cell-cell contact and paracrine signaling, influencing the phenotype and function of innate and adaptive immune cells, while the inflammatory microenvironment reciprocally modulates MSC behavior and differentiation (77, 78).

#### 4.2.1 MSC-induced M2 macrophage polarization

MSC-induced M2 macrophage polarization is a key mechanism by which MSCs promote tissue repair. MSCs secrete soluble factors such as prostaglandin E2 (PGE2), TGF-β, and IL-10, which drive macrophage polarization toward the anti-inflammatory M2 phenotype (8, 76, 78). M2 macrophages, in turn, secrete cytokines and growth factors that support matrix remodeling and regeneration, while reducing pro-inflammatory mediators. This polarization is further enhanced by MSC-derived extracellular vesicles, which can transfer regulatory microRNAs and proteins to macrophages (39, 78).

#### 4.2.2 MSC-mediated suppression of T cell activation and proliferation

MSC-mediated suppression of T cell activation and proliferation occurs via the release of immunomodulatory molecules including indoleamine 2,3-dioxygenase (IDO), nitric oxide, and TGF-β, as well as through direct cell-cell interactions (76, 77). These mechanisms reduce effector T cell responses and promote the expansion of regulatory T cells, thereby dampening local inflammation and preventing immune-mediated tissue damage (1, 42).

#### 4.2.3 Bidirectional effects: inflammatory milieu can inhibit or enhance MSC differentiation

Bidirectional effects of the inflammatory milieu are evident, as acute inflammation can “license” MSCs, enhancing their immunosuppressive and regenerative functions, while chronic or excessive inflammation impairs MSC viability and differentiation potential (76). Pro-inflammatory cytokines such as TNF-α and IFN-γ can inhibit MSC osteogenic and tenogenic differentiation, whereas anti-inflammatory conditions or M2 macrophage presence promote MSC-mediated tissue integration (1, 39).

#### 4.2.4 Secreted factors: PGE2, IDO, TSG-6, HLA-G

Secreted factors including PGE2, IDO, TSG-6, and HLA-G are central to MSC-immune crosstalk. PGE2 and IDO are critical for macrophage polarization and T cell suppression, while TSG-6 and HLA-G contribute to the anti-inflammatory and immunosuppressive milieu that supports tissue regeneration (8, 76, 77). The coordinated action of these factors underpins the therapeutic potential of MSCs in modulating the immune microenvironment for optimal tendon-to-bone healing.

Brief priming with IFN−γ—alone or combined with TNF−α—elevates IL−10, IDO, PGE_2_ and CXCR4/CCR2, thereby strengthening T−cell suppression, Treg/M2 skewing and homing to inflamed sites (79, 80). Inflammation−responsive IL−10 transgenes give a similar context−specific anti−inflammatory boost without constitutive expression (80). These immunoengineering tactics overcome immune barriers in rotator−cuff repair while preserving MSC osteo− and tenogenic potential (81).

### 4.3 Immune-modulatory potentials of MSC-derived extracellular vesicles

Mesenchymal stem cell-derived extracellular vesicles (MSC-EVs) have emerged as critical mediators of immune modulation and tissue regeneration, offering a promising cell-free alternative to traditional MSC-based therapies. Their ability to influence the immune microenvironment is particularly relevant in the context of rotator cuff tendon-to-bone healing, where immune regulation is essential for optimal tissue integration and repair (82, 83).

#### 4.3.1 EVs as cell-free therapeutic tools

MSC-EVs are lipid bilayer-enclosed particles that encapsulate a diverse cargo of proteins, lipids, and nucleic acids, mirroring the therapeutic properties of their parent cells while minimizing risks associated with cell transplantation, such as immune rejection and tumorigenicity (84, 85). Preclinical studies have demonstrated that MSC-EVs can modulate both innate and adaptive immune responses, suppressing pro-inflammatory cytokine production, promoting regulatory T cell expansion, and attenuating macrophage activation (83, 86, 87). These properties are particularly advantageous in musculoskeletal repair, where excessive inflammation impedes tendon-to-bone healing. Additionally, MSC-EVs exhibit favorable pharmacokinetics, low immunogenicity, and the capacity to cross biological barriers, further supporting their translational potential (85, 86, 88). Despite these advantages, challenges remain regarding large-scale production, standardization, and precise targeting of EVs in clinical applications.

#### 4.3.2 Immunomodulatory miRNAs and proteins within EVs

The immunomodulatory effects of MSC-EVs are largely attributed to their cargo of microRNAs (miRNAs) and proteins, which orchestrate complex regulatory networks within recipient immune cells (83, 85, 89). Key miRNAs, such as miR-21, miR-146a, and miR-155, have been shown to downregulate inflammatory signaling pathways (e.g., NF-κB, STAT3), inhibit pro-inflammatory cytokine release, and promote the polarization of macrophages toward an anti-inflammatory (M2) phenotype (86, 87). In addition, MSC-EVs are enriched in immunoregulatory proteins, including TGF-β, HGF, and galectins, which further suppress immune activation and foster a regenerative microenvironment (85, 86). These molecular mediators collectively contribute to the attenuation of local inflammation, enhancement of tissue repair, and prevention of fibrosis in tendon-to-bone healing models (83, 89).

Precisely engineered MSC−derived extracellular vesicles (MSC−EVs) are emerging acellular immunomodulators. Hypoxia, IFN−γ, TNF−α or kartogenin preconditioning enrich cargo (miR−21/146a/155, TGF−β, HGF, galectins), amplifying M2 macrophage polarization, T−reg expansion and NF−κB/STAT3 suppression (90–92). Surface RGD/mannose ligands or SPIO labelling further improve homing to the tendon–bone interface (82, 93). These modifications boost efficacy, cut batch variability and favor scalable “off−the−shelf” translation for rotator−cuff repair (93).

In summary, MSC-EVs represent a versatile and potent cell-free therapeutic platform with robust immunomodulatory capabilities, mediated by their unique repertoire of miRNAs and proteins. Ongoing research is focused on optimizing their production, characterization, and delivery to maximize their clinical utility in musculoskeletal and other regenerative applications.

## 5 Modulation of the immune microenvironment to enhance MSC therapy

**Figure 6:**
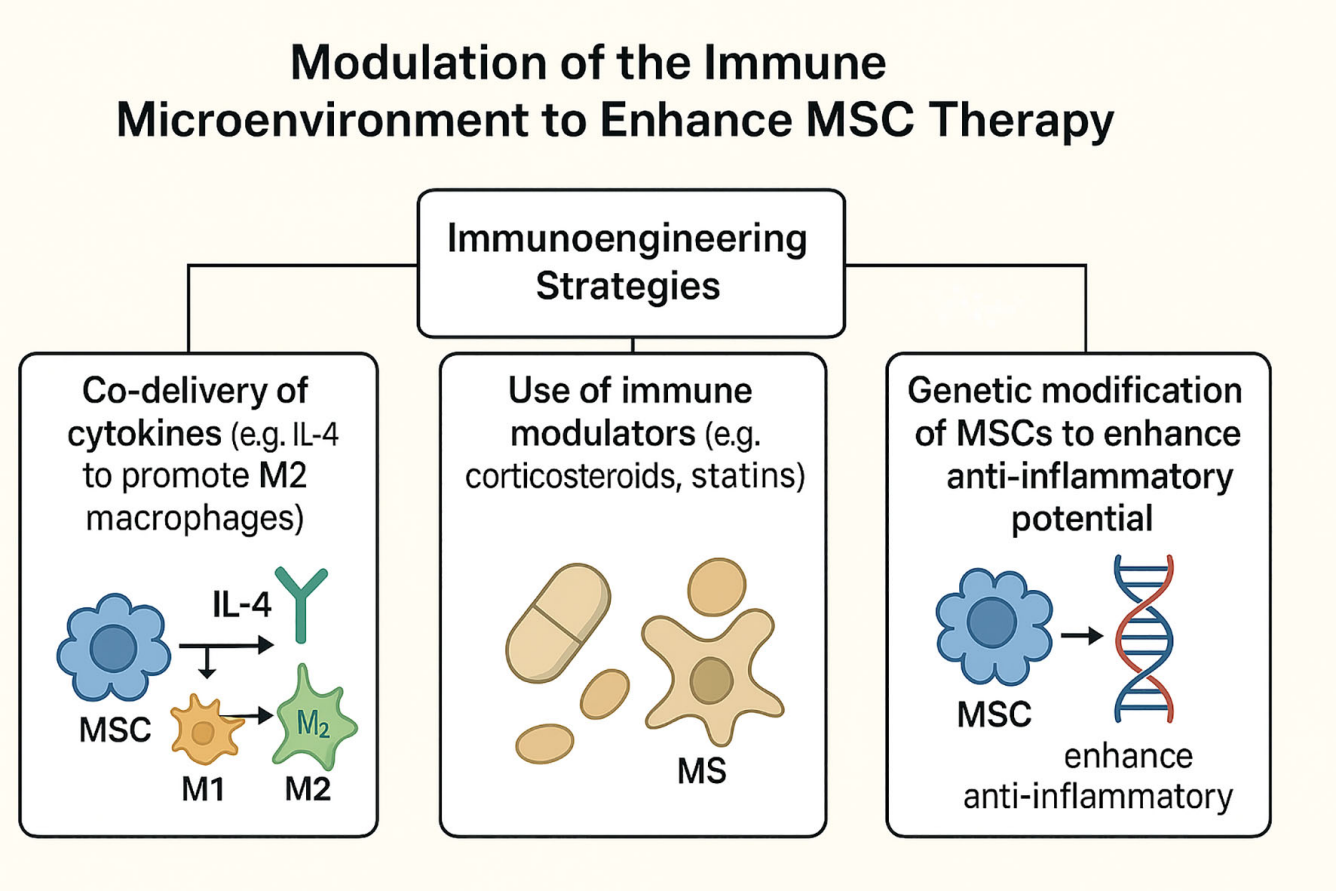
Schematic Overview of Immunoengineering Approaches to Optimize MSC Therapy in Tendon-to-Bone Healing. This diagram illustrates strategies to enhance mesenchymal stem cell (MSC) efficacy by modulating immune interactions, including cytokine co-delivery (e.g., IL-4 to shift macrophages from pro-inflammatory M1 to repair-promoting M2 phenotypes), pharmacological suppression of inflammation via immune modulators (e.g., corticosteroids, statins), and genetic engineering of MSCs to boost anti-inflammatory factor expression. These approaches synergistically improve the immune microenvironment, fostering bidirectional interactions between MSCs and immune cells to accelerate tissue repair and optimize therapeutic outcomes in tendon-to-bone healing.

Modulation of the immune microenvironment is increasingly recognized as a critical determinant of mesenchymal stem cell (MSC) efficacy in rotator cuff tendon-to-bone healing (**Figure 6**). The interplay between MSCs and immune cells, particularly macrophages, orchestrates the inflammatory milieu and subsequent tissue regeneration. Recent studies highlight that targeted immunoengineering strategies can enhance MSC-mediated repair by promoting a pro-regenerative environment and mitigating deleterious inflammation (1, 8, 94).

### 5.1 Immunoengineering strategies

Immunoengineering approaches aim to optimize the local immune context to support MSC survival, engraftment, and paracrine activity. These strategies include the co-delivery of immunomodulatory cytokines, use of pharmacologic agents, and genetic modification of MSCs to augment their anti-inflammatory properties (47, 78, 94, 95).

#### 5.1.1 Co-delivery of cytokines (e.g., IL-4 to promote M2 macrophages)

The co-delivery of cytokines, particularly interleukin-4 (IL-4), has been shown to shift macrophage polarization toward the M2 phenotype, which is associated with anti-inflammatory and tissue-reparative functions. In preclinical models, MSC-derived secretomes and conditioned media have demonstrated the capacity to inhibit pro-inflammatory M1 macrophages and promote M2 polarization, thereby enhancing tendon-bone healing and reducing fibrotic scar formation. The beneficial effects of MSCs in this context are mediated, at least in part, by the regulation of macrophage phenotype via paracrine signaling pathways, including Smad2/3 activation (8, 39, 78).

#### 5.1.2 Use of immune modulators (e.g., corticosteroids, statins)

Pharmacologic agents such as corticosteroids and statins have been investigated for their ability to modulate the immune microenvironment and synergize with MSC therapy (1). These agents can suppress excessive inflammation and promote a milieu conducive to MSC-mediated repair (39). While corticosteroids are well-established anti-inflammatory agents, their use must be balanced against potential inhibitory effects on tissue regeneration (95). Statins, in addition to their lipid-lowering properties, exhibit pleiotropic immunomodulatory effects that may enhance MSC survival and function in the context of tendon healing (47). However, the optimal dosing and timing of these agents in combination with MSCs require further investigation in translational models (96).

#### 5.1.3 Genetic modification of MSCs to enhance anti-inflammatory potential

Genetic engineering of MSCs represents a promising strategy to augment their immunomodulatory and regenerative properties. Approaches include overexpression of anti-inflammatory cytokines, enhancement of paracrine factor secretion, and modification of surface molecules to improve immune evasion and homing (94). Preclinical studies have demonstrated that genetically modified MSCs can more effectively suppress local inflammation, promote M2 macrophage polarization, and facilitate tendon-bone integration (47, 96). The use of exosome-based delivery systems derived from engineered MSCs further amplifies these effects by providing a cell-free platform for targeted immune modulation (96).

Engineering MSCs to express IL−10, TSG−6, HLA−G, HGF or to up−regulate CXCR4/CCR2 augments M2 skewing, T−cell inhibition, angiogenesis and targeted enthesis homing (97, 98)[2−3]. Short IFN−γ priming further elevates IDO activity without safety loss (99, 100)[5−6]. These approaches consistently depress TNF−α/IL−1β, boost IL−10/TGF−β and enhance fibrocartilage strength and load−to−failure outcomes (97, 98)[2−3].

Collectively, these immunoengineering strategies highlight the critical role of modulating the immune microenvironment to fully harness the therapeutic potential of MSCs in rotator cuff tendon-to-bone healing. Continued research is essential to optimize these approaches and facilitate their clinical translation.

### 5.2 Biomaterial-assisted delivery systems

Biomaterial-assisted delivery systems have emerged as a cornerstone in enhancing the therapeutic efficacy of mesenchymal stem cells (MSCs) and immunomodulatory agents for rotator cuff tendon-to-bone healing (101). These platforms address key challenges such as poor cell retention, limited engraftment, and suboptimal control of the local immune microenvironment, which are critical determinants of successful tissue regeneration (102–104).

#### 5.2.1 Hydrogels, scaffolds, microspheres for controlled MSC and cytokine release

Hydrogels, particularly those based on natural or synthetic macromolecules, provide a three-dimensional, extracellular matrix-mimetic environment that supports MSC viability, proliferation, and differentiation while enabling localized, sustained release of bioactive factors (102, 105, 106). Decellularized extracellular matrix (dECM) hydrogels further enhance MSC retention and integration by closely recapitulating native tissue architecture and minimizing immunogenicity (106). Hybrid hydrogels combining dECM with proteins or polysaccharides can further optimize mechanical and biological properties for tendon-to-bone interface repair (106).

Microsphere-containing hydrogels and composite scaffolds allow for precise spatial and temporal control of MSC and cytokine delivery. For example, polylactic-glycolic acid (PLGA) microspheres embedded in hydrogels can sequentially release chemotactic and immunomodulatory peptides, first recruiting endogenous MSCs and then modulating macrophage polarization to favor tissue regeneration (107, 108). Such systems have demonstrated enhanced osteogenesis and improved immune response regulation in preclinical models (107, 108). Electrospun scaffolds, especially when integrated with biological polymers, further improve MSC adhesion, paracrine signaling, and engraftment, supporting robust tissue remodeling (109).

Injectable micro-fragmented nanofiber-hydrogel composites (mfNHCs) have shown promise as minimally invasive carriers for MSC delivery, promoting host macrophage infiltration, pro-regenerative polarization, and angiogenesis in both small and large animal models (110). These advances collectively underscore the importance of biomaterial design in controlling the local microenvironment and optimizing MSC-based therapies for tendon-to-bone healing (101, 102, 108, 109).

#### 5.2.2 Immune-responsive biomaterials to regulate local inflammation

The immune response to implanted biomaterials is a critical determinant of healing outcomes. Recent strategies focus on engineering immune-responsive biomaterials that actively modulate the local inflammatory milieu to promote constructive tissue remodeling (103–105). Hydrogels and scaffolds can be functionalized with immunomodulatory agents or designed to present specific physical and chemical cues that direct macrophage polarization toward a pro-regenerative (M2) phenotype, thereby reducing chronic inflammation and fibrosis (107, 108, 111).

Sequential release systems, such as those delivering LL37 and W9 peptides, exemplify how biomaterials can orchestrate the transition from an initial pro-inflammatory response (necessary for debris clearance and cell recruitment) to a subsequent anti-inflammatory, regenerative phase (107). The physicochemical properties of hydrogels—including crosslinking density, degradation rate, and surface chemistry—can be tuned to modulate immune cell infiltration and phenotype (101, 103, 105, 111). Standardized *in vitro* pathways for evaluating immune responses to biomaterials are being developed to ensure safety and reproducibility in regenerative applications (103, 104).

Engineered MSCs and cargo−enriched EVs—achieved through IL−10/TSG−6/HLA−G or CXCR4/CCR2 overexpression, brief IFN−γ priming, and miRNA−boosting preconditioning—further reinforce M2/T−reg immunity and fibrocartilage formation, but evidence is still largely preclinical and awaits protocol standardization (83, 93).

Overall, incorporating immunomodulatory design principles into biomaterial platforms is crucial for optimizing MSC–immune interactions and improving tendon-to-bone healing outcomes.

### 5.3 Combination therapies

Emerging evidence indicates that combination therapies leveraging mesenchymal stem cells (MSCs) with adjunctive modalities can potentiate tendon-to-bone healing by modulating the immune microenvironment and enhancing tissue regeneration (97, 112, 113). These strategies address the limitations of MSC monotherapy, such as poor engraftment, low survival, and variable efficacy.

#### 5.3.1 MSCs with growth factors or gene therapy

The co-administration of MSCs with growth factors, or genetic modification of MSCs to overexpress trophic factors (e.g., hepatocyte growth factor [HGF]), has been shown to improve cell survival, paracrine signaling, and regenerative capacity in preclinical models (112, 114, 115). HGF gene-modified MSCs, for example, demonstrate enhanced anti-inflammatory and pro-angiogenic effects, which are critical for tendon-bone interface healing (114). Preconditioning MSCs with bioactive substances or optimizing culture conditions further augments their therapeutic potential (97, 115). These approaches are under active investigation for musculoskeletal repair, including tendon injuries (42, 112).

#### 5.3.2 MSCs with regulatory T cells for immune tolerance induction

Combining MSCs with regulatory T cells (Tregs) has shown synergistic immunomodulatory effects, leading to superior attenuation of inflammation compared to either cell type alone (116, 117). In models of traumatic injury, MSC+Treg therapy more effectively suppresses neuroinflammation and modulates systemic immune responses, suggesting potential for improved tendon-to-bone healing by promoting immune tolerance and reducing chronic inflammation (116, 118). This strategy is particularly relevant in settings where immune-mediated tissue damage impedes regeneration.

#### 5.3.3 Synergy with physical rehabilitation protocols

Integrating MSC therapy with structured physical rehabilitation protocols—termed “regenerative rehabilitation”—can enhance functional recovery by promoting graft integration, neural plasticity, and tissue remodeling (112, 119). Rehabilitation modalities may optimize the local microenvironment, facilitating MSC engraftment and paracrine activity (112). Preclinical studies in musculoskeletal and spinal cord injury models support the use of combinatorial regimens to maximize anatomical and functional outcomes (120, 121).

Engineered MSCs combined with regulatory T cells enhance immune tolerance and curb chronic inflammation, but evidence remains limited to animal and early−phase studies (122). Designer EVs enriched with IL−10 or miR−146a suppress inflammation, drive M2/Treg responses, and, when paired with rehabilitation or bFGF−loaded scaffolds, boost angiogenesis and graded mineralization (87, 123). Translation is hampered by dose, delivery, standardization, and safety challenges (93). Advancing MSC-based therapies for rotator cuff repair increasingly relies on integrated strategies, which hold significant promise but require further investigation to establish effective clinical protocols and pathways for translation.

## 6 Current challenges and future directions

### 6.1 Immune and cellular heterogeneity

The principal biological bottleneck is the two−way variability between therapeutic MSCs and the patient’s immune landscape. Source− and donor−dependent heterogeneity (age, sex, metabolic status) produces wide ranges in transcriptomic profile, trophic factor output and immunoregulatory strength (124, 125). Single−cell RNA−seq and proteomics expose discrete MSC sub−clusters with divergent proliferative and anti−inflammatory capacities, explaining batch−to−batch inconsistency (126, 127). Donor−to−donor variation persists even after pooling or pre−treatment strategies (128). On the host side, ageing, diabetes and chronic low−grade inflammation tilt macrophages toward an M1 bias, deplete regulatory T cells and impair enthesis remodelling (129–132). Senescent tendon−stem cells in elderly rotator−cuff tears create a positive pro−inflammatory feedback loop with macrophages that can be broken only by exogenous, rejuvenated EVs38. These intertwined variabilities demand precise MSC characterization, selection of functional sub−populations and immune−responsive licensing—approaches highlighted in Section5 (e.g., IFN−γ priming; IL−10/TSG−6/HLA−G over−expression; cargo−enriched EVs) (79–81, 97–100).

### 6.2 Technical and translational barriers

Regulation & manufacturing. In the UnitedStates, only minimally manipulated same−day autologous MSCs are allowed outside IND trials; expanded products must meet stringent GMP criteria, yet lack universal potency assays (133–135). Variability in cell yield, viability and purity across tissue sources, donors and processing sites hampers multi−center trials and large−scale deployment (1, 134).

Dose, delivery & long−term safety. Optimal cell/EV dose, timing and route remain undefined. Pulmonary first−pass trapping and rapid systemic clearance reduce bioavailability, whereas ectopic bone or fibrotic nodules—though uncommon—raise safety concerns (73, 125, 129). Smart hydrogels with ROS− or MMP−cleavable linkers (Section5.2) prolong local retention, while CXCR4/CCR2 up−regulation enhances homing to inflamed enthesis (101, 107). Long−term monitoring is still needed to rule out tumourigenicity or aberrant differentiation (125, 136).

Protocol & endpoint standardization. Heterogeneous isolation, expansion and storage methods, as well as disparate imaging (MRI vs ultrasound) and biomechanical read−outs, impede meta−analysis (95, 134, 137). Quantitative MRI−T2* and circulating EV signatures are being explored as early, reproducible surrogate markers of tendon−to−bone healing (138–140).

### 6.3 Strategic innovations

#### 6.3.1 Personalized immunomodulatory MSC therapy

Advances in single−cell transcriptomics and multiplex cytokine profiling enable patient immune stratification and MSC sub−typing (74, 141). Matching low−inflammatory MSC subsets to hyper−inflammatory patients—or licensing cells exvivo with IFN−γ to normalize IL−10/IDO output—could minimize heterogeneity and maximize benefit (79–81).

#### 6.3.2 Organoid & 3D−printed enthesis models

Humanized organoids and bioprinted constructs now recreate tendon–bone zonal ECM, permitting high−throughput screening of MSC–immune–biomaterial interactions (142–144). However, current 3D−bioprinted enthesis models still fail to reproduce gradient mineralization, zonal load transfer and anisotropic tendon−bone integration (145–147). Fabricating multiphasic scaffolds with precise spatial control over ECM, stiffness and mineral composition remains technically difficult, impeding functional replication of native enthesis and slowing clinical translation (142, 148).

Consequently, these unresolved biomechanical gaps underscore that present 3D-bioprinted enthesis constructs continue to face substantial mechanical constraints. These include inadequate reproduction of gradient mineralization, impaired zonal load transfer, and insufficient anisotropic integration between tendon and bone tissues (145–147). Moreover, fabricating multiphasic scaffolds with precise spatial organization of ECM, stiffness, and mineral content remains technically difficult (142, 148). These challenges continue to hinder the functional replication of native enthesis and represent major barriers to the clinical translation of 3Dbioprinting in tendon−to−bone repair.

#### 6.3.3 Systems biology & AI−guided prediction tools

Integrating single−cell transcriptomes, cytokine arrays and proteomics with machine−learning enables prediction of MSC potency, identification of immune subtypes linked to poor healing and optimization of donor selection and licensing parameters (149, 150). These AI−derived signatures promise to reduce therapeutic variability and guide personalized immuno−engineering strategies, advancing data −driven precision therapy (151, 152).

Artificial−intelligence tools offer promising means to integrate high−dimensional immune data—such as single−cell transcriptomics, cytokine profiles, and proteomics—for predictive modeling and personalized MSC therapy optimization (152, 153). By uncovering complex patterns across omics datasets, AI algorithms can stratify patients, predict MSC potency, and identify immune subtypes linked to poor healing (154, 155). These approaches also guide donor selection, cell processing, and immunomodulatory strategies, helping to reduce therapeutic variability and enhance clinical outcomes in tendon−to−bone repair.

Together, these emerging strategies tackle cellular−immune heterogeneity and translational barriers, complementing the immuno−engineering and biomaterial approaches detailed earlier. Their convergence is expected to deliver reproducible, patient−tailored MSC/EV therapies for rotator−cuff tendon−to−bone repair.

## 7 Conclusion

Tendon-to-bone healing after rotator cuff repair remains a major clinical challenge due to the limited regenerative capacity of the enthesis and a complex immune microenvironment. MSCs offer promising therapeutic potential through their regenerative and immunomodulatory functions. However, their efficacy is highly dependent on immune interactions at the repair site. Advancements in biomaterial delivery, immunoengineering, and cell-free approaches have improved outcomes in preclinical models. Future strategies that incorporate personalized immune profiling, humanized testing platforms, and AI-driven optimization are expected to further enhance the clinical translation and effectiveness of MSC-based therapies.
